# 3-Amino-1-(4-fluoro­phen­yl)-8-meth­oxy-1*H*-benzo[*f*]chromene-2-carbonitrile

**DOI:** 10.1107/S160053681300545X

**Published:** 2013-03-02

**Authors:** Ahmed M. El-Agrody, Mohamed A. Al-Omar, Abd El-Galil E. Amr, Seik Weng Ng, Edward R. T. Tiekink

**Affiliations:** a Chemistry Department, Faculty of Science, King Khalid University, Abha 61413, PO Box 9004, Saudi Arabia; b Chemistry Department, Faculty of Science, Al-Azhar University, Nasr City, Cairo, 11884, Egypt; c Pharmaceutical Chemistry Department, College of Pharmacy, King Saud University, Riyadh 11451, Saudi Arabia; d Drug Exploration & Development Chair (DEDC), College of Pharmacy, King Saud University, Riyadh 11451, Saudi Arabia; e Applied Organic Chemistry Department, National Research Center, Dokki 12622, Cairo, Egypt; f Department of Chemistry, University of Malaya, 50603 Kuala Lumpur, Malaysia; g Chemistry Department, Faculty of Science, King Abdulaziz University, PO Box 80203 Jeddah, Saudi Arabia

## Abstract

The title compound, C_21_H_15_FN_2_O_2_, features an approximately planar 1*H*-benzo[*f*]chromene fused-ring system (r.m.s. deviation for the 14 non-H atoms = 0.052 Å), with the fluoro­benzene ring being almost perpendicular to this [dihedral angle = 85.30 (7) °]. The furan ring has a flattened half-chair conformation, with the methine C atom deviating by 0.132 (2) Å from the plane of the remaining atoms (r.m.s. deviation = 0.0107 Å). In the crystal, inversion dimers are formed *via* pairs of amine–cyano N—H⋯N hydrogen bonds. The dimers are connected into a three-dimensional architecture by C—H⋯N(cyano), C—H⋯π and π–π [inter­centroid distance = 3.6671 (10) Å] inter­actions.

## Related literature

For background and various applications of benzo- and naphtho­pyran- derivatives, see: Bonsignore *et al.* (1993 ▶); Hafez *et al.* (1987 ▶). For background to the chemistry and biological activity of 4*H*-pyran derivatives, see: El-Agrody *et al.* (2011 ▶); Sabry *et al.* (2011 ▶). For related structures, see: Wang *et al.* (2008 ▶); Shekhar *et al.* (2012 ▶); Amr *et al.* (2013 ▶).
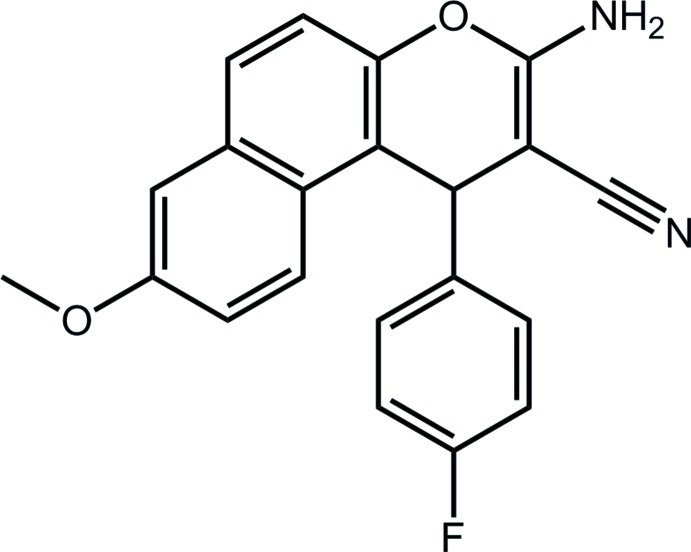


## Experimental

### 

#### Crystal data

C_21_H_15_FN_2_O_2_
*M* *_r_* = 346.35
Triclinic, 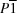

*a* = 8.9672 (7) Å
*b* = 10.4365 (8) Å
*c* = 10.9058 (8) Å
α = 103.063 (7)°
β = 106.859 (7)°
γ = 111.399 (8)°
*V* = 844.01 (11) Å^3^
*Z* = 2
Mo *K*α radiation
μ = 0.10 mm^−1^
*T* = 295 K
0.30 × 0.20 × 0.10 mm

#### Data collection

Agilent SuperNova Dual diffractometer with an Atlas detector
Absorption correction: multi-scan (*CrysAlis PRO*; Agilent, 2011 ▶) *T*_min_ = 0.722, *T*_max_ = 1.000
7109 measured reflections
3902 independent reflections
2716 reflections with *I* > 2σ(*I*)
*R*_int_ = 0.023

#### Refinement

*R*[*F* ^2^ > 2σ(*F* ^2^)] = 0.049
*wR*(*F* ^2^) = 0.138
*S* = 1.05
3902 reflections
244 parameters
H atoms treated by a mixture of independent and constrained refinement
Δρ_max_ = 0.20 e Å^−3^
Δρ_min_ = −0.16 e Å^−3^

### 

Data collection: *CrysAlis PRO* (Agilent, 2011 ▶); cell refinement: *CrysAlis PRO*; data reduction: *CrysAlis PRO*; program(s) used to solve structure: *SHELXS97* (Sheldrick, 2008 ▶); program(s) used to refine structure: *SHELXL97* (Sheldrick, 2008 ▶); molecular graphics: *ORTEP-3 for Windows* (Farrugia, 2012 ▶) and *DIAMOND* (Brandenburg, 2006 ▶); software used to prepare material for publication: *publCIF* (Westrip, 2010 ▶).

## Supplementary Material

Crystal structure: contains datablock(s) global, I. DOI: 10.1107/S160053681300545X/hb7047sup1.cif

Structure factors: contains datablock(s) I. DOI: 10.1107/S160053681300545X/hb7047Isup2.hkl

Supplementary material file. DOI: 10.1107/S160053681300545X/hb7047Isup3.cml

Additional supplementary materials:  crystallographic information; 3D view; checkCIF report

## Figures and Tables

**Table 1:** Hydrogen-bond geometry (Å, °) *Cg*1 is the centroid of the C1–C4,C9,C10 ring.

*D*—H⋯*A*	*D*—H	H⋯*A*	*D*⋯*A*	*D*—H⋯*A*
N1—H1⋯N2^i^	0.89 (2)	2.16 (2)	3.043 (2)	170.0 (18)
C19—H19⋯N2^ii^	0.93	2.51	3.259 (3)	138
C11—H11⋯*Cg*1^iii^	0.98	2.90	3.7084 (17)	141
C14—H14*C*⋯*Cg*1^iv^	0.96	2.92	3.772 (3)	148

Symmetry codes: (i) 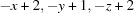; (ii) 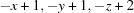; (iii) 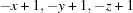; (iv) 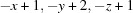.

**Fig. 1.**
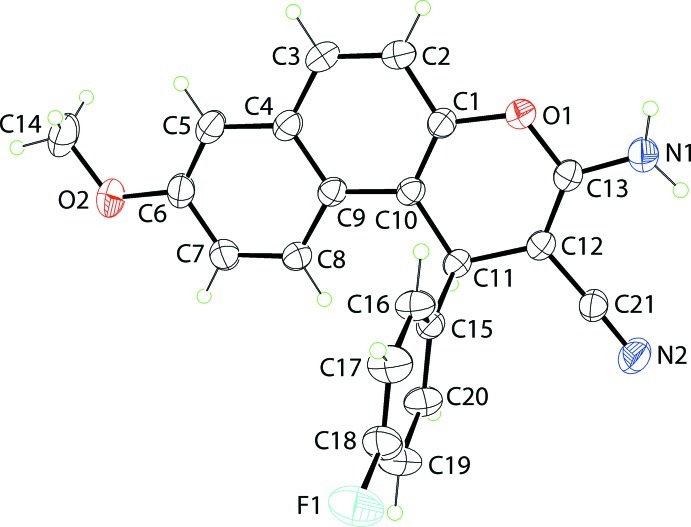
The molecular structure of (I) showing displacement ellipsoids at the 35% probability level.

**Fig. 2.**
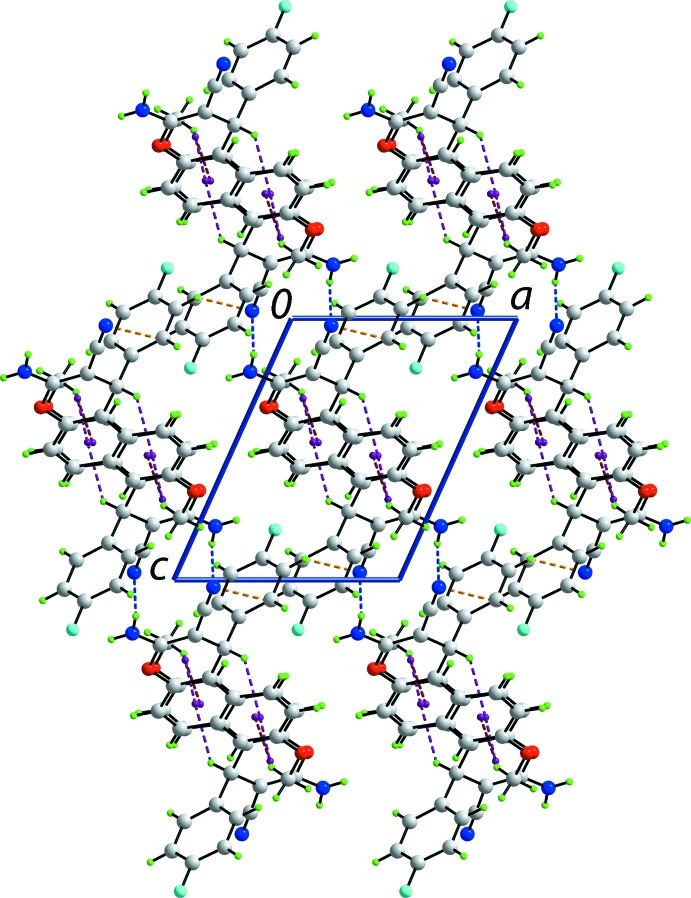
A view in projection down the b axis of the crystal packing in (I). The N—H···N, C—H···N, C—H···π and π–π interactions are shown as blue, orange, purple and brown dashed lines, respectively.

## supplementary crystallographic information

### Comment

Benzo- and naphthopyran-derivatives can possess biological and pharmacological activities, such as anti-coagulant, spasmolytic, diuretic, anti-cancer and anti-anaphylactin activities (Bonsignore *et al.*, 1993). Some of these compounds can also be employed as cosmetics, pigments and as potential biodegradable agrochemicals (Hafez, *et al.*, 1987). Derivatives of 4*H*-pyran are also known to exhibit biological activities (El-Agrody *et al.*, 2011; Sabry *et al.*, 2011), and form the focus of on-going studies of their chemistry. In this connection, herein, the crystal and molecular structure of the title compound, (I), is described.

In (I), Fig. 1, the 14 non-hydrogen atoms of the 1*H*-benzo[*f*]chromene fused-ring system are co-planar with a r.m.s. deviation of the fitted atoms = 0.052 Å. The maximum deviations are 0.136 (2) Å for the methine-C11 atom and -0.052 (2) Å for the aromatic-C7 atom. This implies that the furan ring is approximately planar as borne out by the observation that the methine-C11 atom lies only 0.132 (2) Å above the plane of the remaining atoms (r.m.s. deviation = 0.0107 Å), indicating a flattened half-chair conformation. The fluorobenzene ring is approximately perpendicular to the 1*H*-benzo[*f*]chromene residue, forming a dihedral angle of 85.30 (7)°. The methoxy group is co-planar with the ring to which it is attached as seen in the value of the C14—O2—C6—C5 torsion angle of 0.5 (3)°. The structure described here resembles very closely those of the 4-bromo (Wang *et al.*, 2008) and 2-CF_3_ (Shekhar *et al.*, 2012) derivatives of the parent compound, as well as that of the 7-methoxy analogue (Amr *et al.*, 2013).

Hydrogen bonding features in the crystal packing whereby 12-membered {···HNC_3_N}_2_ synthons formed by centrosymmetrically related molecules arise from the formation of amine-*N*—*H*···*N*(cyano) hydrogen bonds, Table 1. These are connected into a three-dimensional architecture by *C*—*H*···N2(cyano), C—H···π and π—π [inter-centroid distance for (O1,C1,C10–C13)···(C4–C9)^i^ = 3.6671 (10) Å, inter-planar angle = 4.19 (8)° for *i*: 1 - *x*, 1 - *y*, 1 - *z*] interactions, Fig. 2 and Table 1. The second amine-H2 atom does not participate in a significant intermolecular interaction.

### Experimental

A solution of 6-bromo-2-naphthol (0.01 mol) in EtOH (30 ml) was treated with α-cyano-*p*-fluorocinnamonitrile (0.01 mol) and piperidine (0.5 ml). The reaction mixture was heated until complete precipitation occurred corresponding to a reaction time of 60 min. The solid product which formed was collected by filtration and recrystallized from ethanol to give the title compound, (I), in the form of light brown prisms; *M*.pt: 529–530 K.

### Refinement

The C-bound H atoms were geometrically placed (C–H = 0.93–0.98 Å) and refined as riding with *U_iso_*(H) = 1.2*U_eq_*(C). The N-bound-H atoms were refined freely.

### Crystal data

**Table:**

C_21_H_15_FN_2_O_2_	*Z* = 2
*M**_r_* = 346.35	*F*(000) = 360
Triclinic, *P*1	*D*_x_ = 1.363 Mg m^−^^3^
Hall symbol: -P 1	Mo *K*α radiation, λ = 0.71073 Å
*a* = 8.9672 (7) Å	Cell parameters from 2078 reflections
*b* = 10.4365 (8) Å	θ = 3.2–27.5°
*c* = 10.9058 (8) Å	µ = 0.10 mm^−^^1^
α = 103.063 (7)°	*T* = 295 K
β = 106.859 (7)°	Prism, light-brown
γ = 111.399 (8)°	0.30 × 0.20 × 0.10 mm
*V* = 844.01 (11) Å^3^	

### Data collection

**Table:**

Agilent SuperNova Dual diffractometer with an Atlas detector	3902 independent reflections
Radiation source: SuperNova (Mo) X-ray Source	2716 reflections with *I* > 2σ(*I*)
Mirror monochromator	*R*_int_ = 0.023
Detector resolution: 10.4041 pixels mm^-1^	θ_max_ = 27.6°, θ_min_ = 3.2°
ω scan	*h* = −11→11
Absorption correction: multi-scan (*CrysAlis PRO*; Agilent, 2011)	*k* = −13→12
*T*_min_ = 0.722, *T*_max_ = 1.000	*l* = −14→13
7109 measured reflections	

### Refinement

**Table:**

Refinement on *F*^2^	Secondary atom site location: difference Fourier map
Least-squares matrix: full	Hydrogen site location: inferred from neighbouring sites
*R*[*F*^2^ > 2σ(*F*^2^)] = 0.049	H atoms treated by a mixture of independent and constrained refinement
*wR*(*F*^2^) = 0.138	*w* = 1/[σ^2^(*F*_o_^2^) + (0.0568*P*)^2^ + 0.1033*P*] where *P* = (*F*_o_^2^ + 2*F*_c_^2^)/3
*S* = 1.05	(Δ/σ)_max_ < 0.001
3902 reflections	Δρ_max_ = 0.20 e Å^−^^3^
244 parameters	Δρ_min_ = −0.16 e Å^−^^3^
0 restraints	Extinction correction: *SHELXL97* (Sheldrick, 2008), Fc^*^=kFc[1+0.001xFc^2^λ^3^/sin(2θ)]^-1/4^
Primary atom site location: structure-invariant direct methods	Extinction coefficient: 0.031 (4)

### Special details

**Table:**

Geometry. All e.s.d.'s (except the e.s.d. in the dihedral angle between two l.s. planes) are estimated using the full covariance matrix. The cell e.s.d.'s are taken into account individually in the estimation of e.s.d.'s in distances, angles and torsion angles; correlations between e.s.d.'s in cell parameters are only used when they are defined by crystal symmetry. An approximate (isotropic) treatment of cell e.s.d.'s is used for estimating e.s.d.'s involving l.s. planes.
Refinement. Refinement of *F*^2^ against ALL reflections. The weighted *R*-factor *wR* and goodness of fit *S* are based on *F*^2^, conventional *R*-factors *R* are based on *F*, with *F* set to zero for negative *F*^2^. The threshold expression of *F*^2^ > σ(*F*^2^) is used only for calculating *R*-factors(gt) *etc*. and is not relevant to the choice of reflections for refinement. *R*-factors based on *F*^2^ are statistically about twice as large as those based on *F*, and *R*- factors based on ALL data will be even larger.

### Fractional atomic coordinates and isotropic or equivalent isotropic displacement parameters (Å^2^)

**Table:**

	*x*	*y*	*z*	*U*_iso_*/*U*_eq_	
F1	0.65107 (18)	1.02622 (15)	1.19074 (13)	0.0888 (4)	
O1	0.93475 (14)	0.70759 (13)	0.65915 (12)	0.0489 (3)	
O2	0.08769 (17)	0.77609 (16)	0.33736 (14)	0.0685 (4)	
N1	1.0769 (2)	0.62403 (19)	0.79544 (18)	0.0538 (4)	
N2	0.8096 (2)	0.4809 (2)	0.97164 (18)	0.0655 (5)	
C1	0.7901 (2)	0.72705 (17)	0.59527 (16)	0.0416 (4)	
C2	0.8067 (2)	0.7931 (2)	0.49837 (18)	0.0511 (4)	
H2A	0.9069	0.8184	0.4806	0.061*	
C3	0.6756 (2)	0.8201 (2)	0.43063 (18)	0.0518 (4)	
H3	0.6879	0.8664	0.3681	0.062*	
C4	0.5202 (2)	0.77860 (17)	0.45373 (15)	0.0428 (4)	
C5	0.3816 (2)	0.80453 (18)	0.38152 (17)	0.0489 (4)	
H5	0.3935	0.8522	0.3199	0.059*	
C6	0.2310 (2)	0.75979 (19)	0.40231 (17)	0.0493 (4)	
C7	0.2135 (2)	0.6892 (2)	0.49622 (17)	0.0503 (4)	
H7	0.1100	0.6585	0.5094	0.060*	
C8	0.3452 (2)	0.66482 (18)	0.56843 (16)	0.0444 (4)	
H8	0.3307	0.6182	0.6305	0.053*	
C9	0.50492 (19)	0.70961 (16)	0.55037 (15)	0.0391 (4)	
C10	0.64573 (19)	0.68629 (16)	0.62547 (15)	0.0370 (3)	
C11	0.63936 (18)	0.62664 (16)	0.73856 (15)	0.0370 (3)	
H11	0.5315	0.5327	0.7006	0.044*	
C12	0.79503 (19)	0.59719 (16)	0.78873 (15)	0.0396 (4)	
C13	0.93049 (19)	0.63973 (17)	0.75081 (16)	0.0411 (4)	
C14	0.0952 (3)	0.8443 (3)	0.2389 (2)	0.0773 (6)	
H14A	−0.0123	0.8498	0.2013	0.116*	
H14B	0.1107	0.7867	0.1661	0.116*	
H14C	0.1921	0.9424	0.2829	0.116*	
C15	0.63823 (18)	0.73347 (16)	0.85967 (15)	0.0376 (3)	
C16	0.7538 (2)	0.88289 (19)	0.91135 (18)	0.0509 (4)	
H16	0.8299	0.9174	0.8704	0.061*	
C17	0.7591 (3)	0.9820 (2)	1.0222 (2)	0.0614 (5)	
H17	0.8370	1.0825	1.0558	0.074*	
C18	0.6474 (2)	0.9291 (2)	1.08140 (18)	0.0567 (5)	
C19	0.5336 (2)	0.7830 (2)	1.03607 (19)	0.0614 (5)	
H19	0.4600	0.7495	1.0792	0.074*	
C20	0.5293 (2)	0.6848 (2)	0.92405 (17)	0.0507 (4)	
H20	0.4517	0.5844	0.8918	0.061*	
C21	0.80450 (19)	0.53183 (18)	0.88874 (17)	0.0453 (4)	
H1	1.096 (3)	0.588 (2)	0.861 (2)	0.065 (6)*	
H2	1.158 (3)	0.672 (2)	0.772 (2)	0.063 (6)*	

### Atomic displacement parameters (Å^2^)

**Table:**

	*U*^11^	*U*^22^	*U*^33^	*U*^12^	*U*^13^	*U*^23^
F1	0.1109 (10)	0.0868 (9)	0.0662 (8)	0.0463 (7)	0.0490 (7)	0.0064 (7)
O1	0.0466 (6)	0.0623 (7)	0.0539 (7)	0.0294 (5)	0.0262 (5)	0.0343 (6)
O2	0.0705 (8)	0.0905 (10)	0.0680 (9)	0.0550 (8)	0.0249 (7)	0.0441 (8)
N1	0.0463 (9)	0.0631 (10)	0.0626 (10)	0.0293 (7)	0.0230 (7)	0.0338 (8)
N2	0.0573 (9)	0.0839 (12)	0.0741 (11)	0.0350 (8)	0.0293 (8)	0.0542 (10)
C1	0.0456 (8)	0.0443 (9)	0.0396 (8)	0.0234 (7)	0.0177 (7)	0.0188 (7)
C2	0.0538 (10)	0.0611 (11)	0.0502 (10)	0.0272 (8)	0.0289 (8)	0.0301 (9)
C3	0.0615 (10)	0.0584 (11)	0.0460 (9)	0.0287 (8)	0.0257 (8)	0.0306 (8)
C4	0.0527 (9)	0.0411 (9)	0.0357 (8)	0.0234 (7)	0.0165 (7)	0.0155 (7)
C5	0.0637 (11)	0.0474 (10)	0.0396 (9)	0.0299 (8)	0.0178 (8)	0.0203 (7)
C6	0.0575 (10)	0.0528 (10)	0.0426 (9)	0.0345 (8)	0.0152 (8)	0.0176 (8)
C7	0.0516 (9)	0.0617 (11)	0.0456 (9)	0.0328 (8)	0.0210 (8)	0.0210 (8)
C8	0.0515 (9)	0.0515 (10)	0.0376 (8)	0.0281 (7)	0.0201 (7)	0.0194 (7)
C9	0.0466 (8)	0.0376 (8)	0.0320 (8)	0.0205 (6)	0.0146 (6)	0.0115 (6)
C10	0.0445 (8)	0.0350 (8)	0.0318 (7)	0.0190 (6)	0.0153 (6)	0.0124 (6)
C11	0.0380 (8)	0.0355 (8)	0.0364 (8)	0.0154 (6)	0.0142 (6)	0.0157 (6)
C12	0.0442 (8)	0.0377 (8)	0.0383 (8)	0.0195 (6)	0.0159 (6)	0.0168 (7)
C13	0.0443 (8)	0.0390 (8)	0.0399 (8)	0.0205 (7)	0.0150 (7)	0.0156 (7)
C14	0.0899 (15)	0.0878 (16)	0.0666 (13)	0.0557 (13)	0.0184 (11)	0.0427 (12)
C15	0.0386 (8)	0.0412 (8)	0.0351 (8)	0.0187 (6)	0.0145 (6)	0.0181 (6)
C16	0.0589 (10)	0.0453 (10)	0.0502 (10)	0.0188 (8)	0.0305 (8)	0.0192 (8)
C17	0.0745 (12)	0.0440 (10)	0.0576 (11)	0.0199 (9)	0.0313 (10)	0.0123 (9)
C18	0.0660 (11)	0.0633 (12)	0.0422 (9)	0.0335 (9)	0.0254 (8)	0.0117 (9)
C19	0.0593 (11)	0.0734 (13)	0.0491 (10)	0.0220 (9)	0.0328 (9)	0.0195 (9)
C20	0.0501 (9)	0.0492 (10)	0.0450 (9)	0.0129 (7)	0.0224 (7)	0.0172 (8)
C21	0.0399 (8)	0.0488 (9)	0.0491 (9)	0.0212 (7)	0.0159 (7)	0.0234 (8)

### Geometric parameters (Å, º)

**Table:**

F1—C18	1.362 (2)	C8—C9	1.423 (2)
O1—C13	1.3508 (19)	C8—H8	0.9300
O1—C1	1.3933 (18)	C9—C10	1.428 (2)
O2—C6	1.3665 (19)	C10—C11	1.508 (2)
O2—C14	1.419 (2)	C11—C12	1.513 (2)
N1—C13	1.345 (2)	C11—C15	1.529 (2)
N1—H1	0.89 (2)	C11—H11	0.9800
N1—H2	0.87 (2)	C12—C13	1.351 (2)
N2—C21	1.146 (2)	C12—C21	1.410 (2)
C1—C10	1.369 (2)	C14—H14A	0.9600
C1—C2	1.400 (2)	C14—H14B	0.9600
C2—C3	1.356 (2)	C14—H14C	0.9600
C2—H2A	0.9300	C15—C20	1.379 (2)
C3—C4	1.416 (2)	C15—C16	1.381 (2)
C3—H3	0.9300	C16—C17	1.379 (2)
C4—C9	1.415 (2)	C16—H16	0.9300
C4—C5	1.418 (2)	C17—C18	1.363 (3)
C5—C6	1.364 (2)	C17—H17	0.9300
C5—H5	0.9300	C18—C19	1.357 (3)
C6—C7	1.402 (2)	C19—C20	1.387 (2)
C7—C8	1.361 (2)	C19—H19	0.9300
C7—H7	0.9300	C20—H20	0.9300
			
C13—O1—C1	118.99 (12)	C10—C11—C15	111.81 (12)
C6—O2—C14	117.43 (15)	C12—C11—C15	109.77 (12)
C13—N1—H1	120.1 (13)	C10—C11—H11	108.5
C13—N1—H2	114.8 (13)	C12—C11—H11	108.5
H1—N1—H2	123.1 (19)	C15—C11—H11	108.5
C10—C1—O1	122.98 (14)	C13—C12—C21	118.53 (14)
C10—C1—C2	123.20 (15)	C13—C12—C11	123.71 (14)
O1—C1—C2	113.82 (13)	C21—C12—C11	117.57 (13)
C3—C2—C1	119.46 (15)	N1—C13—O1	110.25 (14)
C3—C2—H2A	120.3	N1—C13—C12	127.35 (16)
C1—C2—H2A	120.3	O1—C13—C12	122.39 (14)
C2—C3—C4	120.94 (16)	O2—C14—H14A	109.5
C2—C3—H3	119.5	O2—C14—H14B	109.5
C4—C3—H3	119.5	H14A—C14—H14B	109.5
C9—C4—C5	120.25 (15)	O2—C14—H14C	109.5
C9—C4—C3	118.64 (15)	H14A—C14—H14C	109.5
C5—C4—C3	121.11 (16)	H14B—C14—H14C	109.5
C6—C5—C4	120.17 (16)	C20—C15—C16	118.03 (15)
C6—C5—H5	119.9	C20—C15—C11	121.98 (14)
C4—C5—H5	119.9	C16—C15—C11	119.93 (13)
O2—C6—C5	125.58 (17)	C17—C16—C15	121.56 (16)
O2—C6—C7	114.43 (15)	C17—C16—H16	119.2
C5—C6—C7	119.99 (15)	C15—C16—H16	119.2
C8—C7—C6	121.13 (16)	C18—C17—C16	118.34 (17)
C8—C7—H7	119.4	C18—C17—H17	120.8
C6—C7—H7	119.4	C16—C17—H17	120.8
C7—C8—C9	120.94 (16)	C19—C18—F1	118.80 (17)
C7—C8—H8	119.5	C19—C18—C17	122.38 (16)
C9—C8—H8	119.5	F1—C18—C17	118.82 (17)
C4—C9—C10	120.40 (14)	C18—C19—C20	118.59 (16)
C4—C9—C8	117.51 (14)	C18—C19—H19	120.7
C10—C9—C8	122.09 (15)	C20—C19—H19	120.7
C1—C10—C9	117.30 (14)	C15—C20—C19	121.09 (16)
C1—C10—C11	121.40 (14)	C15—C20—H20	119.5
C9—C10—C11	121.23 (13)	C19—C20—H20	119.5
C10—C11—C12	109.72 (12)	N2—C21—C12	177.89 (18)
			
C13—O1—C1—C10	3.0 (2)	C8—C9—C10—C11	−6.2 (2)
C13—O1—C1—C2	−177.54 (13)	C1—C10—C11—C12	−9.55 (19)
C10—C1—C2—C3	0.5 (3)	C9—C10—C11—C12	173.70 (12)
O1—C1—C2—C3	−179.02 (15)	C1—C10—C11—C15	112.49 (15)
C1—C2—C3—C4	−1.7 (3)	C9—C10—C11—C15	−64.26 (17)
C2—C3—C4—C9	0.7 (3)	C10—C11—C12—C13	9.0 (2)
C2—C3—C4—C5	−179.04 (16)	C15—C11—C12—C13	−114.21 (16)
C9—C4—C5—C6	−1.6 (2)	C10—C11—C12—C21	−176.02 (13)
C3—C4—C5—C6	178.06 (15)	C15—C11—C12—C21	60.74 (17)
C14—O2—C6—C5	0.5 (3)	C1—O1—C13—N1	176.64 (13)
C14—O2—C6—C7	−178.87 (16)	C1—O1—C13—C12	−3.7 (2)
C4—C5—C6—O2	−178.78 (15)	C21—C12—C13—N1	1.8 (3)
C4—C5—C6—C7	0.6 (3)	C11—C12—C13—N1	176.66 (15)
O2—C6—C7—C8	179.82 (15)	C21—C12—C13—O1	−177.78 (14)
C5—C6—C7—C8	0.4 (3)	C11—C12—C13—O1	−2.9 (2)
C6—C7—C8—C9	−0.3 (3)	C10—C11—C15—C20	137.00 (15)
C5—C4—C9—C10	−178.61 (13)	C12—C11—C15—C20	−100.99 (17)
C3—C4—C9—C10	1.7 (2)	C10—C11—C15—C16	−45.73 (19)
C5—C4—C9—C8	1.6 (2)	C12—C11—C15—C16	76.27 (18)
C3—C4—C9—C8	−178.05 (14)	C20—C15—C16—C17	−1.3 (3)
C7—C8—C9—C4	−0.7 (2)	C11—C15—C16—C17	−178.64 (16)
C7—C8—C9—C10	179.57 (14)	C15—C16—C17—C18	0.4 (3)
O1—C1—C10—C9	−178.74 (13)	C16—C17—C18—C19	0.8 (3)
C2—C1—C10—C9	1.8 (2)	C16—C17—C18—F1	−179.78 (17)
O1—C1—C10—C11	4.4 (2)	F1—C18—C19—C20	179.49 (17)
C2—C1—C10—C11	−175.07 (14)	C17—C18—C19—C20	−1.1 (3)
C4—C9—C10—C1	−2.9 (2)	C16—C15—C20—C19	1.0 (3)
C8—C9—C10—C1	176.87 (14)	C11—C15—C20—C19	178.28 (16)
C4—C9—C10—C11	174.03 (13)	C18—C19—C20—C15	0.2 (3)

### Hydrogen-bond geometry (Å, º)

Cg1 is the centroid of the C1–C4,C9,C10 ring.

**Table:**

*D*—H···*A*	*D*—H	H···*A*	*D*···*A*	*D*—H···*A*
N1—H1···N2^i^	0.89 (2)	2.16 (2)	3.043 (2)	170.0 (18)
C19—H19···N2^ii^	0.93	2.51	3.259 (3)	138
C11—H11···*Cg*1^iii^	0.98	2.90	3.7084 (17)	141
C14—H14*C*···*Cg*1^iv^	0.96	2.92	3.772 (3)	148

Symmetry codes: (i) −*x*+2, −*y*+1, −*z*+2; (ii) −*x*+1, −*y*+1, −*z*+2; (iii) −*x*+1, −*y*+1, −*z*+1; (iv) −*x*+1, −*y*+2, −*z*+1.
